# Profilaxia da trombose venosa profunda em cirurgia bariátrica: estudo comparativo com doses diferentes de heparina de baixo peso molecular

**DOI:** 10.1590/1677-5449.008417

**Published:** 2018

**Authors:** Carlos José Goslan, Giórgio Alfredo Pedroso Baretta, Hemuara Grasiela Pestana de Souza, Bruna Zanin Orsi, Esdras Camargo A. Zanoni, Marco Antonio Gimenez Lopes, Carlos Alberto Engelhorn

**Affiliations:** 1 Angiolab Curitiba, Laboratório Vascular Não Invasivo, Curitiba, PR, Brasil.; 2 Hospital Vita Batel, Curitiba, PR, Brasil.; 3 Pontifícia Universidade Católica do Paraná – PUCPR, Curitiba, PR, Brasil.

**Keywords:** profilaxia, trombose venosa, obesidade

## Abstract

**Contexto:**

A cirurgia bariátrica é considerada a melhor opção para o tratamento da obesidade, cujos pacientes são considerados de alto risco para fenômenos tromboembólicos.

**Objetivos:**

Comparar o uso de doses diferentes de heparina de baixo peso molecular (HBPM) na profilaxia da trombose venosa profunda (TVP) em pacientes candidatos à cirurgia bariátrica em relação ao risco de TVP, alteração na dosagem do fator anti-Xa e sangramento pré ou pós-operatório.

**Métodos:**

Estudo comparativo transversal em pacientes submetidos à cirurgia bariátrica distribuídos em dois grupos, que receberam doses de HBPM de 40 mg (grupo controle, GC) e 80 mg (grupo de estudo, GE). Foram avaliados por ultrassonografia vascular e dosagem de KPTT, TAP, plaquetas e fator anti-Xa.

**Resultados:**

Foram avaliados 60 pacientes, sendo 34 no GC e 26 no GE. Foi observada diferença significativa somente no peso (p = 0,003) e índice de massa corporal (p = 0,018) no GE em relação ao GC. Não houve diferença na dosagem de KPTT, TAP, plaquetas e fator anti-Xa entre os grupos. Não foram detectados TVP ou sangramentos significativos em ambos os grupos.

**Conclusões:**

Não houve diferença estatisticamente significativa na utilização de doses maiores de HBPM na profilaxia da TVP em pacientes candidatos à cirurgia bariátrica em relação ao risco de TVP, dosagem do fator anti-Xa e sangramento pré ou pós-operatório.

## INTRODUÇÃO

 A obesidade, atualmente, é considerada uma epidemia global e representa um problema de saúde de incidência crescente 1 ^-^ 3 . São 600 milhões de pessoas portadoras dessa doença no mundo, sendo que 5% delas encontram-se no Brasil e são responsáveis por cerca de 10% dos gastos de saúde pública 1 . O risco de mortalidade pela obesidade aumenta entre 6 e 12 vezes em relação à população normal hígida, e a expectativa de vida é reduzida em 12 anos para homens e 9 anos para mulheres 4 . É uma doença caracterizada pelo acúmulo excessivo de gordura no organismo, gerando um estado inflamatório crônico 1 . Quando o gasto energético é inferior à oferta de calorias, o resultado é um aumento ponderal associado, frequentemente, a danos à saúde 5 . 

 O tecido adiposo libera grande quantidade de fator de necrose tumoral alfa (TNF-α) e interleucina 6 (IL-6) e ativa neutrófilos, reduzindo a capacidade imunológica do indivíduo. Além disso, há hipercoagulabilidade devido à alteração na concentração do fibrinogênio, ao aumento do inibidor do ativador do plasminogênio 1 (PAI-1) e à diminuição de antitrombina III e da fibrinólise. Há ainda alterações metabólicas como hiperinsulinemia (pelo aumento da resistência à insulina), aumento da retenção renal de sódio, ativação do sistema nervoso simpático, dislipidemia, hiperuricemia e intolerância à glicose 1 . 

 Progressos têm sido alcançados no tratamento farmacológico da obesidade mórbida, porém o método cirúrgico tem sido considerado mais eficaz, além de ser capaz de controlar as comorbidades causadas pelo aumento da gordura corporal 1 ^-^ 3 ^,^ 6 . As indicações clássicas para a cirurgia bariátrica são: índice de massa corporal (IMC) > 40 kg/m^2^ ou IMC > 35 kg/m^2^ na presença de comorbidades ocasionadas ou agravadas pela obesidade, com pelo menos dois anos de evolução. Além disso, os obesos devem ter realizado tratamentos convencionais prévios sem sucesso ou apresentado recidiva do peso 1 ^,^ 3 ^,^ 7 . 

 Tanto a obesidade quanto o seu tratamento cirúrgico são considerados fatores de risco para o desencadeamento de eventos trombóticos. O desenvolvimento do tromboembolismo venoso ocorre em virtude da alteração de um ou mais dos fatores da tríade de Virchow 8 ^,^ 9 . O risco desses eventos é determinado por fatores intrínsecos, que englobam condições pessoais, congênitas ou adquiridas, e por fatores extrínsecos e ambientais 4 . A trombose isolada nas veias musculares da panturrilha ocorre em aproximadamente 28% dos casos e pode se estender para os territórios femoropoplíteo e iliacofemoral 10 . 

 A profilaxia para trombose venosa profunda (TVP) deve ser feita em todo paciente obeso submetido à cirurgia bariátrica, independentemente de sua idade, de acordo com a estratificação de risco e o tipo de anestesia a ser usada. No entanto, devido ao grande ganho ponderal desses pacientes, pode ser que a dosagem de heparina de baixo peso molecular (HBPM) recomendada na literatura seja subestimada, havendo, portanto, a necessidade de comparar a dose recomendada com doses maiores de HBPM 11 . 

 O objetivo deste trabalho é comparar o uso de doses diferentes de HBPM na profilaxia da TVP em pacientes candidatos à cirurgia bariátrica em relação ao risco de TVP, alteração na dosagem do fator anti-Xa e sangramento pré ou pós-operatório. 

## MÉTODOS

 Foi realizado um estudo prospectivo comparativo em pacientes submetidos à cirurgia bariátrica no Hospital Vita Batel no período de maio de 2014 a setembro de 2016. O estudo foi aprovado pelo Comitê de Ética em Pesquisa da Pontifícia Universidade Católica do Paraná (PUCPR), sob o protocolo nº 700.665. 

 Foram incluídos no estudo pacientes maiores de 18 anos, com obesidade graus II e III, risco cirúrgico ASA I a III, submetidos à cirurgia de derivação gástrica. Foram excluídos pacientes com obesidade grau I (exceto se apresentaram comorbidades graves), risco cirúrgico ASA IV, etilismo, alergia a HBPM (enoxaparina) e TVP prévia diagnosticada por exame de imagem. Não foi realizado o cálculo amostral da população incluída no estudo. Foram estudados pacientes consecutivos que preencheram os critérios de inclusão no estudo e assinaram o termo de consentimento. 

**Figura 1:**
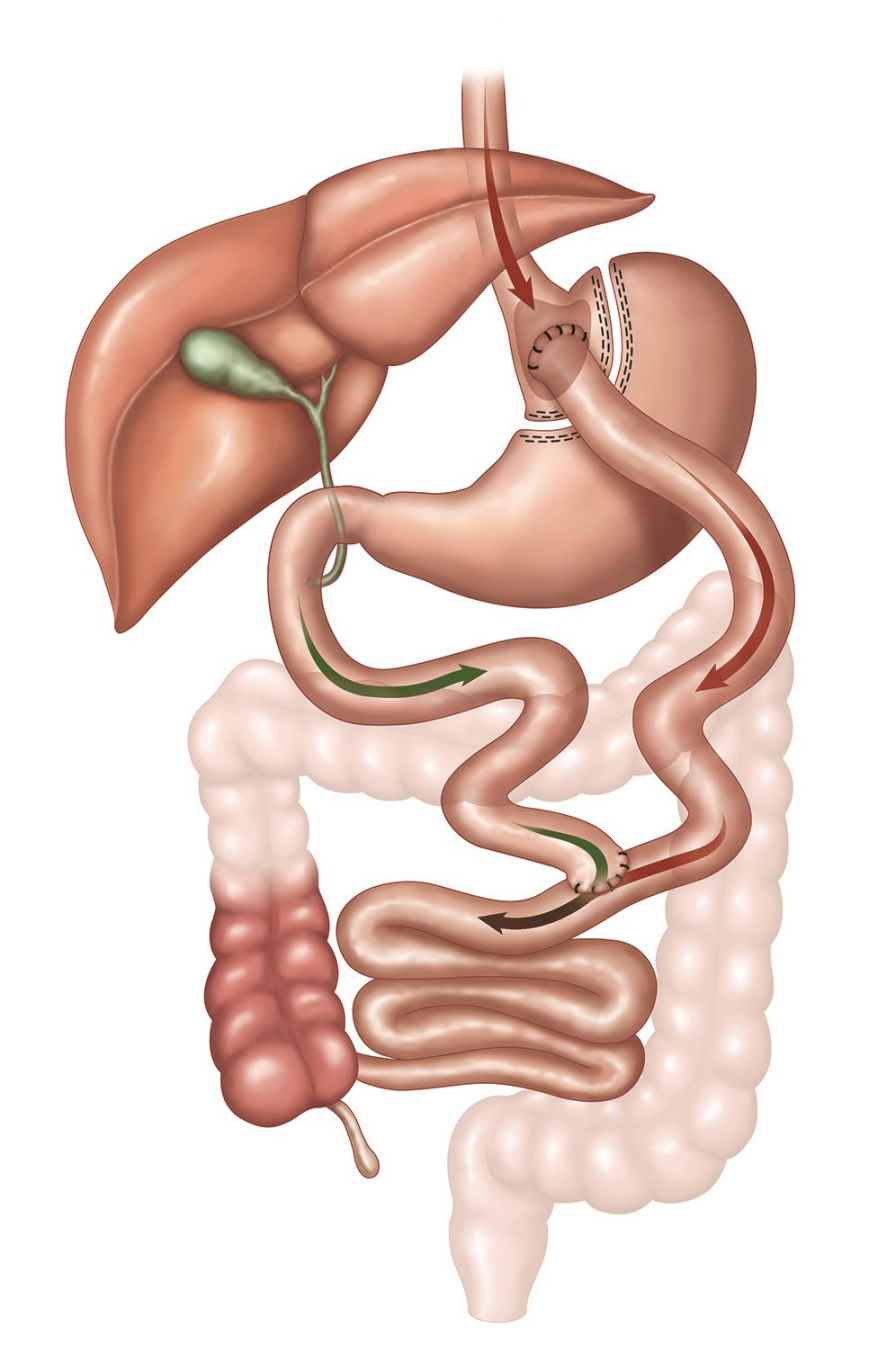
Ilustração do procedimento cirúrgico de *bypass* gástrico com septação gástrica e desvio intestinal. Fonte: Imagem cedida pela Johnson & Johnson do Brasil Indústria e Comércio de Produtos para Saúde Ltda.

 Todos os pacientes incluídos no estudo foram submetidos ao mesmo procedimento cirúrgico de *bypass* gástrico laparoscópico ( Figura 1 ), o qual consiste em uma septação gástrica criando um reservatório de 50 mL e um desvio intestinal de cerca de 200 cm (técnica mista). Foram divididos em dois grupos: grupo controle (GC), que recebeu a dose padrão de profilaxia de 40 mg de enoxaparina uma vez ao dia (primeira dose 12 horas antes do procedimento), e grupo estudo (GE), que recebeu a dose de profilaxia de 40 mg de enoxaparina a cada 12 horas (primeira dose 12 horas antes do procedimento). Ambos os grupos receberam HBPM durante 10 dias no pós-operatório. A seleção dos pacientes para cada grupo foi realizada pelo anestesista antes do procedimento cirúrgico, no momento de estabelecer o acesso venoso, de forma mascarada, por sorteio simples (sem repetições). 

 Todos os pacientes realizaram eco-Doppler venoso (EDV) pré-operatório para investigar TVP no máximo 7 dias antes do procedimento cirúrgico e no décimo dia de pós-operatório. Foram avaliadas rotineiramente as veias femoral comum, femoral profunda, femoral, poplítea, tibiais posteriores, fibulares, musculares gastrocnêmias e musculares soleares, e as veias safenas magna e parva. Os critérios diagnósticos de TVP pelo EDV foram baseados na identificação do trombo intraluminal, incompressibilidade da veia e ausência de fluxo venoso. 

 Em todos os pacientes foi realizada a dosagem sanguínea do fator anti-Xa para avaliar o nível de ação da HBPM no quinto dia de pós-operatório, 4h após a administração da HBPM, com *kit* Berichrom no coagulômetro CS5100 Siemens^R^. Também foi coletada amostra sanguínea para dosagem de plaquetas, TAP e TTPA. Além da profilaxia medicamentosa e deambulação precoce, todos os pacientes usaram botas pneumáticas durante 24h e meia elástica antitrombo (18 mmHg) durante 10 dias no pós-operatório. 

 Os resultados das variáveis quantitativas foram descritos por médias, medianas, valores mínimos, valores máximos e desvios padrão. Variáveis categóricas foram descritas por frequências e percentuais. Para a comparação dos dois grupos com relação a variáveis quantitativas, foi usado o teste *t* de Student para amostras independentes. A condição de normalidade das variáveis foi avaliada pelo teste de Kolmogorov-Smirnov. Com relação a variáveis categóricas, as comparações foram feitas usando o teste exato de Fisher. Para avaliar a associação entre duas variáveis quantitativas, foram estimados os coeficientes de correlação de Pearson. Valores de p < 0,05 indicaram significância estatística. Os dados foram analisados com o programa computacional IBM SPSS Statistics, v. 20. 

## RESULTADOS

 Foram avaliados 67 pacientes submetidos à cirurgia bariátrica, sendo 36 no GC e 31 no GE. Dois pacientes do GC e cinco pacientes do GE não retornaram para a avaliação pós-operatória com EDV. 

 Dos 34 pacientes do GC, havia três homens e 31 mulheres, com idade variando entre 21 e 52 anos (média de 33,3 anos), 14,7% eram diabéticos e 42% hipertensos, com peso variando entre 77 e 149 kg (média de 99,4 kg) e IMC variando entre 32,6 e 45,5 (média de 38,8). Um paciente com IMC inferior a 35 foi incluído devido à indicação cirúrgica pelas comorbidades apresentadas. 

 Dos 26 pacientes do GE, havia quatro homens e 22 mulheres, com idade variando entre 20 e 55 anos (média de 38,8 anos), 19,2% eram diabéticos e 38,5% hipertensos, com peso variando entre 81 e 159 kg (média de 113,1 kg) e IMC variando entre 35,1 e 58,4 (média de 41,7). Todos os pacientes foram submetidos ao mesmo procedimento por videolaparoscopia, com tempo médio de duração de 50 minutos, sem diferença significativa na duração do procedimento entre os grupos estudados. 

 A comparação entre os grupos com relação às características da população ( Tabela 1 ) mostrou diferença estatisticamente significativa somente no peso (p = 0,003) e IMC (p = 0,018) do GE em relação ao GC. Com relação aos exames laboratoriais pré-operatórios, não houve diferença estatisticamente significativa nos valores do colesterol, triglicerídeos e glicemia entre os grupos. 

**Tabela 1:** Comparação entre os grupos controle (GC) e de estudo (GE) com relação a idade, peso e índice de massa corporal (IMC).

**Variável**	**Grupo**	**N**	**Média**	**Mediana**	**Mínimo**	**Máximo**	**Desvio padrão**	**Valor de p** ^*^
Idade	GC	34	33,3	32,5	21	52	8,0	
	GE	26	33,8	33	20	55	9,8	0,821
Peso	GC	34	99,4	96,3	77	149	14,4	
	GE	26	113,1	111	81	159	19,5	0,003
IMC	GC	34	38,8	38,6	32,6	45,5	3,2	
	GE	26	41,7	40,5	35,1	58,4	5,2	0,018

* Teste *t* de Student para amostras independentes, p < 0,05.

 Considerando as dosagens de KPTT, TAP, plaquetas e fator anti-Xa ( Tabela 2 ), não houve diferença estatisticamente significativa nos valores entre os grupos. Especificamente, a dosagem do fator anti-Xa variou entre 0,2 e 0,7 (média de 0,4) no GC e 0,1 e 0,7 (média de 0,4) no GE (p = 0,386). Quanto à correlação da dosagem do fator anti-Xa e o IMC no GC e GE ( Tabela 3 ), observou-se uma fraca correlação (-0,23 e -0,12, respectivamente) em ambos os grupos. 

**Tabela 2:** Comparação entre os grupos controle (GC) e de estudo (GE) com relação às dosagens de fator anti-Xa, TAP, KPPT e plaquetas.

**Variável**	**Grupo**	**n**	**Média**	**Mediana**	**Mínimo**	**Máximo**	**Desvio padrão**	**Valor de p** ^*^
Dosagem anti-Xa 5º PO	GC	34	0,40	0,40	0,20	0,70	0,13	
	GE	26	0,40	0,40	0,10	0,70	0,13	0,386
TAP 5º PO	GC	33	1,10	1,10	0,87	1,20	0,09	
	GE	24	1,10	1,10	1,00	1,20	0,05	0,203
KPTTT 5º PO	GC	33	31,2	29,8	25,9	65,5	6,86	
	GE	24	29,8	29,1	25	37,6	3,09	0,194
Plaquetas 5º PO	GC	33	315948	329000	284	457000	88851	
	GE	24	345542	332500	224000	520000	76135	0,193

* Teste *t* de Student para amostras independentes, p < 0,05.

**Tabela 3:** Correlação entre índice de massa corporal e dosagem do fator anti-Xa.

**Grupo**	**n**	**Coeficiente de correlação de Pearson**	**Valor de p**
Controle	34	-0,23	0,192
Estudo	26	-0,12	0,569

 A comparação de idade, dosagem do fator anti-Xa, TAP, KPTTT e plaquetas entre GC ( Tabela 4 ) e GE ( Tabela 5 ) com IMC inferior e superior a 40 não demonstrou diferença estatisticamente significativa em ambos os grupos. Com relação à presença de sangramento intraoperatório no GC, houve ausência de sangramento significativo. No entanto, no GE ocorreram dois casos de sangramento da linha de grampeamento do estômago excluso facilmente controlado com sobressutura local com PDS 3-0, realizada de rotina em todos os pacientes operados. 

**Tabela 4:** Comparação no grupo controle de idade, dosagem do fator anti-Xa, TAP, KPTT e plaquetas com índice de massa corporal (IMC) inferior e superior a 40.

**Variável**	**IMC**	**n**	**Média**	**Mediana**	**Mínimo**	**Máximo**	**Desvio padrão**	**Valor de p** ^*^
Idade	≤ 40	24	33,5	31,5	21	49	7,7	
	> 40	10	32,8	32,5	21	52	9,3	0,811
Dosagem anti-Xa 5º PO	≤ 40	24	0,40	0,40	0,20	0,60	0,12	
	> 40	10	0,30	0,30	0,20	0,70	0,10	0,222
TAP 5º PO	≤ 40	23	1,10	1,10	0,90	1,20	0,08	
	> 40	10	1,10	1,10	0,87	1,20	0,10	0,566
KPTTT 5º PO	≤ 40	23	31,9	29,8	26	65,5	8	
	> 40	10	29,6	29,3	25,9	35,5	2,7	0,230
Plaquetas 5º PO	≤ 40	23	323304	323000	210000	457000	70852	
	> 40	10	299028	342500	284	426000	123862	0,573

* Teste *t* de Student para amostras independentes, p < 0,05.

**Tabela 5:** Comparação no grupo de estudo de idade, dosagem do fator anti-Xa, TAP, KPTT e plaquetas com índice de massa corporal (IMC) inferior e superior a 40.

**Variável**	**IMC**	**n**	**Média**	**Mediana**	**Mínimo**	**Máximo**	**Desvio padrão**	**Valor de p** ^*^
Idade	≤ 40	10	37,6	36	20	55	10,2	
	> 40	16	31,5	30	20	50	9,0	0,123
Dosagem de anti-Xa 5º PO	≤ 40	10	0,40	0,40	0,10	0,60	0,15	
	> 40	16	0,40	0,40	0,20	0,70	0,12	0,795
TAP 5º PO	≤ 40	10	1,10	1,10	1,00	1,10	0,05	
	> 40	14	1,10	1,10	1,00	1,20	0,05	0,136
KPTTT 5º PO	≤ 40	10	30,1	29,6	25	35,2	3,5	
	> 40	14	29,6	29,1	25,9	37,6	2,9	0,704
Plaquetas 5º PO	≤ 40	10	312400	316500	224000	388000	51721,91	
	> 40	14	369214	346500	270000	520000	83396	0,070

* Teste *t* de Student para amostras independentes, p < 0,05.

 Em ambos os grupos não houve necessidade de conversão da cirurgia videolaparoscópica para cirurgia convencional devido a sangramento ou outras complicações intraoperatórias. No pós-operatório, tanto no GC quanto no GE, dois pacientes apresentaram melena em pequena quantidade, sem nenhuma repercussão hemodinâmica. Com relação à ocorrência de TVP nos membros inferiores, em ambos os grupos não houve identificação de TVP na avaliação pelo EDV pré ou pós-operatória. 

## DISCUSSÃO

 A obesidade está associada a diversas comorbidades causadas, agravadas ou com seu controle prejudicado pelo excesso de peso, incluindo hipertensão arterial, diabetes melito tipo 2, cardiomiopatia, artropatias degenerativas, colelitíase, esteatose hepática, apneia do sono, varizes de membros inferiores, síndrome metabólica e depressão 5 ^,^ 10 ^,^ 11 . É classificada em diversos graus, que permitem uma correlação entre o IMC e os riscos aos quais o indivíduo obeso está propenso. Um IMC entre 25 a 29 kg/m^2^ caracteriza sobrepeso; entre 30 a 34,9 kg/m^2^, obesidade grau I ou leve; entre 35 a 39,9 kg/m^2^, obesidade grau II ou moderada; e maior que 40 kg/m ^2^, grau III ou mórbida 5 ^,^ 10 . 

 Na população incluída no nosso estudo, não foi identificada diferença significativa em idade, sexo, comorbidades e exames laboratoriais pré-operatórios. No entanto, houve diferença estatisticamente significativa no peso e IMC maiores no GE em relação ao GC. 

 As indicações clássicas para a cirurgia bariátrica são IMC > 40 kg/m^2^ ou IMC > 35 kg/m^2^ na presença de comorbidades ocasionadas ou agravadas pela obesidade, com pelo menos dois anos de evolução. A cirurgia pode ser realizada por via aberta ou videolaparoscópica, sendo a segunda mais vantajosa por apresentar uma taxa reduzida de hérnias incisionais e menor tempo de hospitalização. São classificadas de acordo com os seus mecanismos de ação em: restritiva [colocação de banda gástrica ajustável laparoscópica e gastroplastia vertical (*sleeve* )], amplamente restritiva/má absorção discreta (derivação gástrica em Y de Roux) e má absorção intensa/discretamente restritiva [derivação biliopancreática (Scopinaro)] 5 ^,^ 7 ^,^ 12 . 

 A técnica escolhida foi o *bypass* gástrico laparoscópico, consagrado na literatura e o mais realizado em nosso país atualmente. Promove perda de 75-80% do excesso de peso corporal em até 18-24 meses de pós-operatório e melhoria da maioria das comorbidades associadas à obesidade. Essa técnica consiste na septação e redução gástrica para menor ingesta alimentar e saciedade mais precoce e duradoura, além de um desvio intestinal para redução da absorção dos alimentos e alterações hormonais que contribuem para a perda ponderal e melhorias na parte metabólica do paciente. 

 A TVP e o tromboembolismo pulmonar (TEP) são as grandes causas de morbimortalidade pós-cirurgia bariátrica, sendo que um IMC > 40 kg/m^2^ é fator independente para morte súbita por TEP 1 ^,^ 13 ^-^ 15 . Isso é justificado pelo fato de a cirurgia bariátrica ser demorada, necessitando de anestesia com duração superior a 30 minutos, pelos pacientes possuírem condicionamento físico e comorbidades que restringem suas movimentações e pela suspeita de TVP ser mais difícil em obesos mórbidos devido às suas constituições físicas 4 . 

 O TEP é a complicação pós-cirurgia bariátrica mais temida, sendo que idade, IMC > 40 kg/m^2^, gênero masculino e história prévia de TEP são os mais importantes fatores de risco envolvidos. A TVP pode causar morte súbita por embolia pulmonar em 0,3% dos casos de cirurgia bariátrica ou morbidade em longo prazo quando evolui como síndrome pós-trombótica 4 ^,^ 13 . 

 Escalante-Tattersfield et al. 16 realizaram um estudo retrospectivo com 618 pacientes submetidos ao *bypass* gástrico em Y de Roux por laparoscopia e acompanhados por 52 semanas. Todos os pacientes receberam a mesma quantidade de 5000 UI de heparina não fracionada a cada 8h nas primeiras 24h, seguida de 40 mg de enoxaparina a cada 12h, e permaneceram, em média, 4 dias internados. A atelectasia basal foi a complicação hospitalar mais comum (8,4%), seguida de sangramento gastrointestinal (1,6%). Durante todo o acompanhamento, não houve nenhum caso de morte e nenhum paciente apresentou sintomas clínicos de TVP. Apenas um paciente foi diagnosticado com TVP pelo EDV de membros inferiores, realizado nas primeiras 24h após a cirurgia. 

 No nosso estudo, a frequência de IMC > 40 foi de 32% no GC e 59% no GE e, apesar da diferença estatisticamente significativa (p = 0,018), não foi detectada TVP pelo EDV em ambos os grupos. Isso demonstra que a dose recomendada de 40 mg/dia foi capaz de proteger os indivíduos do GC mesmo com obesidade mórbida. Os pacientes obesos são considerados de risco moderado para desenvolver TVP no pós-operatório gástrico quando há um efetivo método profilático e de alto risco quando não há nenhum tipo de profilaxia. O evento trombótico ocorre, em média, após cinco dias da realização da cirurgia 8 . 

 Safdie et al. 17 realizaram um estudo retrospectivo envolvendo 1.503 pacientes que realizaram cirurgia bariátrica, com incidência de 1,3% de TVP no membro superior no período de acompanhamento pós-cirúrgico (30 dias). Nenhum paciente apresentou dispneia, dor pleurítica ou outros sintomas clássicos de TEP. Em um estudo realizado por Prystowsky et al. 18 com 106 pacientes submetidos a cirurgia bariátrica, não houve mortalidade por TEP e foi identificado somente um caso de TVP após 14 dias da cirurgia. Em uma análise do banco de dados nacional dos pacientes internados nos Estados Unidos entre 2007 e 2009, feita por Stein e Matta 19 , em 508.230 cirurgias bariátricas realizadas foi identificada uma incidência de 1,3% de TVP e de 0,9% de TEP, sendo a taxa de mortalidade por TEP de 3,9% naqueles que utilizaram filtros de veia cava e 2,7% nos que não utilizaram. 

 Com relação à profilaxia de eventos tromboembólicos, o American College of Chest Physicians preconiza a profilaxia mecânica, preferencialmente a compressão pneumática intermitente para pacientes de baixo risco. Para os pacientes de moderado risco e sem risco de sangramento, recomenda-se a HBPM ou a heparina não fracionada ou a profilaxia mecânica (compressão pneumática intermitente). Para pacientes de alto risco e sem risco de sangramento, é feita a profilaxia com HBPM ou heparina não fracionada, associada a compressão pneumática intermitente ou a meias elásticas. Se houver contraindicação para o uso de heparina e baixo risco de sangramento, recomenda-se utilizar doses baixas de aspirina, fondaparinux ou profilaxia mecânica (compressão pneumática intermitente) 10 . 

 No nosso estudo, os pacientes foram estratificados como alto risco para desenvolver TVP e, portanto, foi optado pela profilaxia com enoxaparina com dose de 40 mg ao dia. No entanto, devido ao grande ganho ponderal dos pacientes submetidos à cirurgia bariátrica, foi questionado se a dosagem de HBPM recomendada na literatura poderia ser subestimada e se eventualmente haveria necessidade de doses maiores de HBPM para essa população, com eventual risco maior de sangramento pré ou pós-operatório ou de outras complicações. 

 Scholten et al. compararam doses diferentes de enoxaparina (30 mg *versus* 40 mg a cada 12h) em 481 pacientes submetidos a cirurgia bariátrica e identificaram a ocorrência de TVP de 5,4% no grupo de dose menor (30 mg) e de 0,6% no grupo de dose maior (40 mg), com a ressalva de que no grupo de dose menor (30 mg) a duração do procedimento cirúrgico e do tempo de internação foi maior 20 . Borkgren-Okonek et al. 21 utilizaram doses maiores na profilaxia de TVP em 223 pacientes submetidos a *bypass* gástrico, sendo 40 mg a cada 12h nos pacientes com IMC < 50 e 60 mg a cada 12h nos pacientes com IMC maior que 50, e não encontraram diferença significativa na ocorrência de TVP (0,45%) ou sangramento (2,2%). 

 A comparação do uso de doses diferentes de HBPM na profilaxia da TVP no nosso estudo mostrou que, para a população avaliada, não houve incidência de TVP (nos segmentos femoropoplíteo e popliteopodal e nas veias musculares gastrocnêmias e soleares) em nenhum dos grupos avaliados. Contudo, deve-se ressaltar que, além da profilaxia medicamentosa e deambulação precoce, todos os pacientes usaram botas pneumáticas durante 24h e meia elástica antitrombo (18 mmHg) durante 10 dias no pós-operatório. Essas medidas adicionais podem ter influenciado os resultados, mas a adoção delas com a dose padrão de HBPM de acordo com o risco dos pacientes foi suficiente para evitar TVP no pós-operatório. 

 Com relação às complicações intra e pós-operatórias, houve ausência de sangramento significativo intraoperatório no GC, mas, no GE, foram identificados dois casos de sangramento mínimo da linha de grampeamento do estômago excluso, controlado com sucesso com a sobressutura realizada rotineiramente. Apesar de ter ocorrido sangramento de mínimo volume e de fácil controle, o fato de ter ocorrido somente no GE pode estar relacionado à dose maior da HBPM e, por isso, poderia ser considerado uma complicação intraoperatória, com eventual risco maior de sangramento caso seja optado por doses maiores de HBPM na profilaxia da TVP. Em ambos os grupos não houve necessidade de conversão da cirurgia videolaparoscópica para cirurgia convencional devido a sangramento ou outras complicações intraoperatórias. 

 No pós-operatório, tanto no GC quanto no GE, somente dois pacientes apresentaram melena. Isso pode ocorrer naturalmente devido aos resíduos hemáticos após os grampeamentos do estômago e do intestino, além das anastomoses, gerando pequenos sangramentos intraluminais que são expelidos na forma de sangue coagulado misturado às fezes. Além disso, anticoagulantes também podem ser a causa de sangramentos intraluminais que se manifestam dessa maneira no pós-operatório, geralmente autolimitados. 

 Considerando a possibilidade de maior risco de sangramento no GE devido a doses maiores de HBPM em relação ao GC, a comparação entre a dosagem do fator anti-Xa, KPTT, TAP e plaquetas não mostrou diferença estatisticamente significativa entre os grupos, apesar de o GE ter mais pacientes com IMC > 40. 

 A heparina é um anticoagulante composto por cadeias polissacarídeas de diferentes pesos moleculares com grande afinidade com a antitrombina (AT), inibidor fisiológico da coagulação, especialmente trombina (IIa) e fator X ativado (Xa). As HBPMs, com cadeias menores, mantêm sua atividade anti-Xa, mas têm atividade anti-IIa reduzida. O monitoramento da dosagem do fator anti-Xa para guiar a profilaxia é uma opção, apesar de sua utilidade ainda não estar universalmente definida e os estudos controlados não demonstrarem clara relação com sangramento ou eventos tromboembólicos 15 . 

 A recomendação é medir o pico de atividade anti-Xa aproximadamente 4h após o uso de HBPM subcutânea, sendo que cada tipo de HBPM apresenta níveis diferentes de concentração. Tanto para profilaxia quanto para tratamento foi medida a ação anti-Xa das HBPMs em voluntários, sendo que a enoxaparina, droga utilizada em nosso estudo, foi testada em indivíduos de até 120 kg. A maioria dos autores realizou a dosagem do fator anti-Xa entre o terceiro e o quinto dia pós-operatório 22 . 

 A média da atividade anti-Xa, mensurada 3 a 5 horas após a injeção SC, é proporcional à dose administrada, correspondendo a 0,2, 0,4, 1,0 e 1,3 UI anti-Xa/mL nas doses únicas de 20, 40, 1 e 1,5 mg/kg, respectivamente 23 ^-^ 25 . Levine et al. demonstraram correlação estatística entre os níveis de fator anti-Xa inferiores a 0,1 UI/mL com eventos tromboembólicos 26 . 

 Quanto à correlação da dosagem do fator anti-Xa e o IMC no GC e no GE, no nosso estudo observou-se uma fraca correlação em ambos os grupos. A dosagem do fator anti-Xa não apresentou diferença significativa entre os grupos, variando entre 0,2 e 0,7 (média de 0,4) no GC e entre 0,1 e 0,7 (média de 0,4) no GE, sendo que em ambos os grupos a média ponderal foi inferior a 120 kg. 

 Os autores concluem, portanto, que não houve diferença estatisticamente significativa na utilização de doses maiores de HBPM na profilaxia da TVP em pacientes candidatos à cirurgia bariátrica em relação a risco de TVP, dosagem do fator anti-Xa e sangramento pré ou pós-operatório. 
