# Supplementary material for: Profilaxia da trombose venosa profunda em cirurgia bariátrica: estudo comparativo com doses diferentes de heparina de baixo peso molecular
Source: J Vasc Bras. 2018 Jan-Mar;17(1):26–33. [Article in Portuguese] doi: 10.1590/1677-5449.008417 (PMC5990259; doi:10.1590/1677-5449.008417)
Supplement: Translation

# Translation: Deep venous thrombosis prevention in bariatric surgery: comparative study of different doses of low weight molecular heparin

**Authors:** Carlos José Goslan, Giórgio Alfredo Pedroso Baretta, Hemuara Grasiela Pestana de Souza, Bruna Zanin Orsi, Esdras Camargo A. Zanoni, Marco Antonio Gimenez Lopes, Carlos Alberto Engelhorn

**Affiliations:** 1 Angiolab Curitiba, Laboratório Vascular Não Invasivo, Curitiba, PR, Brasil.; 2 Hospital Vita Batel, Curitiba, PR, Brasil.; 3 Pontifícia Universidade Católica do Paraná – PUCPR, Curitiba, PR, Brasil.

**Keywords:** prevention, venous thrombosis, obesity

## Abstract

**Background:**

Bariatric surgery is considered the best treatment option for patients with obesity who are classed as high risk for thromboembolic events.

**Objectives:**

To compare two different doses of low weight molecular heparin (LWMH) for prevention of deep venous thrombosis (DVT) in candidates for bariatric surgery, in terms of DVT risk, abnormal anti-Xa levels, and preoperative and/or postoperative bleeding.

**Methods:**

A cross-sectional comparative study of bariatric surgery patients divided into two groups given different doses of LWMH; 40 mg of LWMH (control group, CG) and 80 mg of LWMH (study group, SG), both evaluated by vascular ultrasonography (VU) and according to the results of PTT, PT, platelets, and anti-Xa factor assays.

**Results:**

Sixty patients were evaluated, 34 in the CG and 26 in the SG. The only significant differences between the patients in the SG and the CG were weight (p = 0.003) and body mass index (p = 0.018). There were no differences between the groups in PTT, PT, platelets, or anti-Xa factor levels. There was no DVT or significant bleeding in either group.

**Conclusions:**

There were no statistical differences when higher doses of LWMH were used for prevention of DVT in bariatric surgery patients, in terms of DVT risk, anti-Xa factor levels, or preoperative and postoperative bleeding.

## INTRODUCTION

 Obesity is now considered a global epidemic and is a health problem with growing incidence. 1 ^-^ 3 There are 600 million people with this disease in the world and 5% of them are in Brazil and are responsible for around 10% of public health spending. 1 Risk of mortality among the obese increases from 6 to 12 times in relation to the normal healthy population and life expectancy is reduced by 12 years in men and 9 years in women. 4 The disease is characterized by excessive accumulation of fat in the body, which causes a chronic inflammatory state. 1 When energy expenditure is lower than the supply of calories, the result is weight increase, frequently associated with damage to health. 5

 Adipose tissue releases large quantities of tumor necrosis factor alpha (TNF-α) and interleukin 6 (IL-6) and activates neutrophils, reducing a person’s immunological capacity. Additionally, there is also hypercoagulability because of abnormal fibrinogen concentration, increased plasminogen activator inhibitor 1 (PAI-1), and reduced antithrombin III and fibrinolysis. There are also metabolic abnormalities such as hyperinsulinemia (due to increased insulin resistance), increase renal sodium retention, activation of the sympathetic nervous system, dyslipidemia, hyperuricemia, and glucose intolerance. 1

 Progress has been achieved in pharmacological treatment of morbid obesity, but the surgical method is considered more effective and is also capable of controlling comorbidities caused by increased body fat. 1 ^-^ 3 ^,^ 6 The classical indications for bariatric surgery are: body mass index (BMI) > 40 kg/m ^2^ or BMI > 35 kg/m^2^ in the presence of comorbidities caused or aggravated by obesity that have been present for at least two years. Additionally, obese candidates must have already undergone conventional treatments previously, either without success or followed by recurrent increase in weight. 1 ^,^ 3 ^,^ 7

 Obesity itself and surgery to treat it are both considered risk factors for thrombotic events. Development of venous thromboembolism is triggered by a change to one or more of the factors that make up Virchow’s triad. 8 ^,^ 9 The risk of these events is determined by intrinsic factors that include personal, congenital, or acquired conditions and extrinsic and environmental conditions. 4 Isolated thrombosis of the muscular calf veins occurs in approximately 28% of cases and can extend to the femoropopliteal, iliac, and femoral territories. 10

 Prophylaxis to prevent deep venous thrombosis (DVT) should be administered to all obese patients who undergo bariatric surgery, irrespective of age, and should be chosen according to risk stratification and the type of anesthesia that will be used. However, because of the considerable weight these patients gain, it is possible that the low molecular weight heparin (LWMH) dosages recommended in the literature are underestimated, hence the need to compare the recommended dose with larger doses of LWMH. 11

 The objective of this study is to compare two different LWMH dosages for DVT prophylaxis in patients who are candidates for bariatric surgery, in terms of risk of DVT, abnormal anti-Xa levels, and preoperative and/or postoperative bleeding 

## METHODS

 A prospective, comparative study was conducted with patients who underwent bariatric surgery at the Hospital Vita Batel, Curitiba, PR, Brazil, from May 2014 to September 2016. The study was approved by the Research Ethics Committee at the Pontifícia Universidade Católica do Paraná, under protocol number 700.665. 

 Patients were enrolled on the study if they were over the age of 18 years, had obesity grade II or III, ASA surgical risk I to III, and underwent gastric derivation surgery. Patients were excluded if they had grade I obesity (except if they had serious comorbidities), ASA grade IV surgical risk, alcoholism, allergy to LWMH (enoxaparin), or previous DVT diagnosed by an imaging exam. No sample size calculation was performed for the population enrolled on the study. Consecutive patients who met the inclusion criteria and signed the consent form were enrolled. 

 All of the patients in the study underwent the same surgical procedure for laparoscopic gastric bypass ( Figure 1 ), consisting of a gastric partitioning to construct a 50 mL pouch and an intestinal bypass of around 200 cm (mixed technique). The patients were assigned to two groups: a control group (CG), given the standard prophylaxis dosage of 40 mg of enoxaparin once a day (first dose 12 hours before the procedure), and a study group (SG), given a 40mg enoxaparin prophylaxis dose every 12 hours (first dose 12 hours before the procedure). Both groups were given LWMH for 10 days during the postoperative period. Patients were assigned to groups by the anesthetist, before the surgical procedure, at the point that venous access was achieved, blinded and by simple lots (without repetitions). 

**Figure 1:** Illustration of surgical procedure for gastric bypass with gastric partitioning and intestinal deviation. Source: Image provided by Johnson & Johnson do Brasil Indústria e Comércio de Produtos para Saúde Ltda.

 All patients underwent preoperative venous Doppler ultrasonography (VDU) to investigate DVT a maximum of 7 days before the surgical procedure and on the tenth day after surgery. All examinations assessed the common femoral, deep femoral, femoral, popliteal, posterior tibial, fibular, gastrocnemius and soleal muscle veins and the great and small saphenous veins. The criteria for diagnosing DVT by VDU were based on identification of intraluminal thrombus, incompressibility of the vein, and absence of venous flow. 

 Anti-Xa factor was assayed in blood from all patients to assess the level of LWMH effect on the fifth day after surgery, 4h after administration of LWMH, using a Berichrom kit in a Siemens CS5100 coagulation monitor. Blood samples were also taken for platelet, PT, and PTT assays. In addition to pharmaceutical prophylaxis and early mobilization, all patients wore pneumatic boots for 24h and antithrombotic compression stockings (18 mmHg) for 10 days during the postoperative period. 

 Results for quantitative variables are expressed as means, medians, minimum and maximum values, and standard deviations. Categorical variables were expressed as frequencies and percentages. Student’s *t* test for independent samples was used to compare quantitative variables between the two groups. The Kolmogorov-Smirnov test was used to assess normality of variables. Fisher’s exact test was used to compare categorical variables. Pearson correlation coefficients were estimated to assess associations between quantitative variables. P values < 0.05 indicated statistical significance. Data were analyzed using IBM SPSS Statistics, v. 20. 

## RESULTS

 A total of 67 patients who had bariatric surgery were analyzed; 36 in the CG and 31 in the SG. Two patients in the CG and five patients in the SG did not return for the postoperative examination with VDU. 

 The remaining 34 patients in the CG were three men and 31 women, with ages ranging from 21 to 52 years (mean of 33.3 years); 14.7% had diabetes and 42% had hypertension, body weight ranged from 77 to 149 kg (mean of 99.4 kg), and BMI ranged from 32.6 to 45.5 (mean of 38.8). One patient with BMI below 35 was included because comorbidities justified surgery. 

 The remaining 26 patients in the SG were four men and 22 women, with age ranging from 20 to 55 years (mean of 38.8 years); 19.2% had diabetes and 38.5% had hypertension, body weight ranged from 81 to 159 kg (mean of 113.1 kg), and BMI ranged from 5.1 to 58.4 (mean of 41.7). All patients were treated by videolaparoscopy using the same procedure, with mean duration of 50 minutes and no significant difference in duration of procedure between the study groups. 

 Comparison of the groups in terms of the characteristics of the population ( Table 1 ) only revealed statistically significant differences between the SG and the CG in weight (p = 0.003) and BMI (p = 0.018). There were no statistically significant differences between groups in terms of preoperative laboratory test results for cholesterol, triglycerides, or glycemia. 

**Table 1:** Comparison of control group (CG) and study group (SG) by age, weight, and body mass index (BMI).

**Variable**	**Group**	**N**	**Mean**	**Median**	**Minimum**	**Maximum**	**Standard deviation**	**P value** ^*^
Age	CG	34	33.3	32.5	21	52	8.0	
	SG	26	33.8	33	20	55	9.8	0.821
Weight	CG	34	99.4	96.3	77	149	14.4	
	SG	26	113.1	111	81	159	19.5	0.003
BMI	CG	34	38.8	38.6	32.6	45.5	3.2	
	SG	26	41.7	40.5	35.1	58.4	5.2	0.018

* Student’s *t* test for independent samples, p < 0.05.

 There were no statistically significant differences between groups in the results of PTT, PT, platelets, or anti-Xa factor assays ( Table 2 ). Anti-Xa factor levels in particular ranged from 0.2 to 0.7 (mean of 0.4) in the CG and from 0.1 to 0.7 (mean of 0.4) in the SG (p = 0.386). Coefficients for the correlations between anti-Xa factor and BMI in the CG and the SG ( Table 3 ) indicated a weak correlation (-0.23 and -0.12, respectively) in both groups. 

**Table 2:** Comparison of control group (CG) and study group (SG) by results for anti-Xa factor, PT, PPT, and platelets.

**Variable**	**Group**	**n**	**Mean**	**Median**	**Minimum**	**Maximum**	**Standard deviation**	**P value** ^*^
Anti-Xa, day 5 PO	CG	34	0.40	0.40	0.20	0.70	0.13	
	SG	26	0.40	0.40	0.10	0.70	0.13	0.386
PT, day 5 PO	CG	33	1.10	1.10	0.87	1.20	0.09	
	SG	24	1.10	1.10	1.00	1.20	0.05	0.203
PTT, day 5 PO	CG	33	31.2	29.8	25.9	65.5	6.86	
	SG	24	29.8	29.1	25	37.6	3.09	0.194
Platelets, day 5 PO	CG	33	315948	329000	284	457000	88851	
	SG	24	345542	332500	224000	520000	76135	0.193

* Student’s *t* test for independent samples, p < 0.05.

**Table 3:** Correlation between body mass index and anti-Xa factor level.

**Group**	**n**	**Pearson’s correlation coefficient**	**P value**
Control	34	-0.23	0.192
Study	26	-0.12	0.569

 Intra-group comparisons of age, anti-Xa factor level, PT, PTT, and platelets between patients with BMI less than 40 and patients with BMI of 40 or more did not reveal statistically significant differences in the CG ( Table 4 ) or the SG ( Table 5 ). With regard to intraoperative bleeding, there was no significant bleeding in the CG. In the SG, however, there were two cases of bleeding along the line of staples for stomach exclusion that were easily controlled with local tie-over PDS 3-0 sutures, which was performed as routine in all of the patients operated on. 

**Table 4:** Comparison in control group of age, anti-Xa factor level, PT, PTT and platelets, by body mass index (BMI) below or above 40.

**Variable**	**BMI**	**n**	**Mean**	**Median**	**Minimum**	**Maximum**	**Standard deviation**	**P value** ^*^
Age	≤ 40	24	33.5	31.5	21	49	7.7	
	> 40	10	32.8	32.5	21	52	9.3	0.811
Anti-Xa, day 5 PO	≤ 40	24	0.40	0.40	0.20	0.60	0.12	
	> 40	10	0.30	0.30	0.20	0.70	0.10	0.222
PT, day 5 PO	≤ 40	23	1.10	1.10	0.90	1.20	0.08	
	> 40	10	1.10	1.10	0.87	1.20	0.10	0.566
PTT, day 5 PO	≤ 40	23	31.9	29.8	26	65.5	8	
	> 40	10	29.6	29.3	25.9	35.5	2.7	0.230
Platelets, day 5 PO	≤ 40	23	323304	323000	210000	457000	70852	
	> 40	10	299028	342500	284	426000	123862	0.573

* Student’s *t* test for independent samples, p < 0.05.

**Table 5:** Comparison in study group of age, anti-Xa factor level, PT, PTT and platelets, by body mass index (BMI) below or above 40.

**Variable**	**BMI**	**n**	**Mean**	**Median**	**Minimum**	**Maximum**	**Standard deviation**	**P value** ^*^
Age	≤ 40	10	37.6	36	20	55	10.2	
	> 40	16	31.5	30	20	50	9.0	0.123
Anti-Xa, day 5 PO	≤ 40	10	0.40	0.40	0.10	0.60	0.15	
	> 40	16	0.40	0.40	0.20	0.70	0.12	0.795
PT, day 5 PO	≤ 40	10	1.10	1.10	1.00	1.10	0.05	
	> 40	14	1.10	1.10	1.00	1.20	0.05	0.136
PTT, day 5 PO	≤ 40	10	30.1	29.6	25	35.2	3.5	
	> 40	14	29.6	29.1	25.9	37.6	2.9	0.704
Platelets, day 5 PO	≤ 40	10	312400	316500	224000	388000	51721.91	
	> 40	14	369214	346500	270000	520000	83396	0.070

* Student’s *t* test for independent samples, p < 0.05.

 There were no cases in either group in which it was necessary to change from videolaparoscopic surgery to conventional surgery because of bleeding or other intraoperative complications. During the postoperative period, two patients in each group exhibited small quantities of melena, with no hemodynamic repercussions. With relation to lower limb DVT, no cases of DVT were identified during preoperative or postoperative VDU examinations. 

## DISCUSSION

 Obesity is associated with many comorbidities that are either caused by, aggravated by, or harder to control because of excess weight. These include arterial hypertension, type 2 Diabetes mellitus, cardiomyopathy, degenerative arthropathies, cholelithiasis, hepatic steatosis, sleep apnea, lower limb varicose veins, metabolic syndrome, and depression. 5 ^,^ 10 ^,^ 11 Obesity can be classified by grades that correlate BMI to the risk to which obese people are prone A BMI from 25 to 29 kg/m^2^ is defined as overweight; from 30 to 34.9 kg/m^2^ is grade I, or mild, obesity; from 35 to 39.9 kg/m^2^ is grade II, or moderate, obesity; and BMI greater than 40 kg/m^2^ is grade III, or morbid, obesity. 5 ^,^ 10

 In the population enrolled on our study there were no significant differences in age, sex, comorbidities, or preoperative laboratory tests. However, there were statistically significant differences in weight and BMI, which were greater in the SG than in the CG. 

 The classical indications for bariatric surgery are BMI > 40 kg/m^2^ or BMI > 35 kg/m^2^ in conjunction with comorbidities caused or aggravated by obesity, present for at least 2 years. Surgery can be open or conducted via videolaparoscopy. The latter is preferable because it is associated with a lower rate of incisional hernias and shorter hospital stays. Procedures are classified according to their mechanisms of action as: restriction (laparoscopic adjustable gastric banding and vertical “sleeve” gastroplasty), intense restriction/discrete malabsorption (Roux-en-Y gastric bypass) and intense malabsorption/discrete restriction (“Scopinaro” biliopancreatic diversion). 5 ^,^ 7 ^,^ 12

 The technique chosen was laparoscopic gastric bypass, which is supported by the literature and is currently the most widely used method in Brazil. It provokes loss of 75-80% of excess body weight in 18-24 months after the operation and improvements in the majority of the comorbidities associated with obesity. The technique consists of gastric partitioning and reduction, to reduce food intake and provoke earlier and longer-lasting satiety, in addition to intestinal diversion to reduce absorption of nutrients and effect hormonal changes that contribute to weight loss and improve metabolic aspects. 

 The greatest causes of morbidity and mortality after bariatric surgery are DVT and pulmonary thromboembolism (PTE) and BMI > 40 kg/m^2^ is an independent risk factor for sudden death from PTE. 1 ^,^ 13 ^-^ 15 This is because of the long duration of bariatric surgery, demanding anesthesia for more than 30 minutes, because patients are in a physical condition and have comorbidities that restrict their movements, and because suspicion of DVT is less likely among the morbidly obese because of their physical constitutions. 4

 The most severe complication after bariatric surgery is PTE and age, BMI > 40 kg/m^2^ , male sex, and prior history of PTE are the most important risk factors involved. In turn, DVT can cause sudden death from pulmonary embolism in 0.3% of bariatric surgery cases or long term morbidity when it leads to postthrombotic syndrome. 4 ^,^ 13

 Escalante-Tattersfield et al. 16 conducted a retrospective study with 618 patients who underwent Roux-en-Y Gastric Bypass by laparoscopy and were followed-up for 52 weeks. All patients were given the same quantity (5000 UI) of unfractionated heparin every 8h for the first 24h, followed by 40 mg of enoxaparin every 12h, and remained in hospital for a mean stay of 4 days. Basal atelectasis was the most common complication while in hospital (8.4%), followed by gastrointestinal bleeding (1.6%). There were no deaths during follow-up and none of the patients exhibited clinical symptoms of DVT. Just one patient was diagnosed with DVT by VDU of the lower limbs, conducted within the first 24h after surgery. 

 In our study, the frequency of BMI > 40 was 32% in the CG and 59% in the SG and, despite this statistically significant difference (p = 0.018), no cases of DVT were detected by VDU in either group. This demonstrates that the recommended dose of 40 mg/day was sufficient to protect the patients in the CG group, even those who were morbidly obese. Obese patients are considered at moderate risk of developing DVT during the postoperative period after gastric surgery when given an effective prophylactic method and at high risk in the absence of any type of prophylaxis. On average, the thrombotic event occurs 5 days after surgery. 8

 Safdie et al. 17 conducted a retrospective study involving 1,503 patients who underwent bariatric surgery, with a 1.3% incidence of DVT in upper limbs during the post-surgical follow-up period (30 days). None of the patients exhibited dyspnea, pleuritic pain, or other classic symptoms of PTE. In a study conducted by Prystowsky et al., 18 analyzing 106 patients who underwent bariatric surgery, there was zero mortality from PTE and only one DVT case was identified, 14 days after surgery. Stein and Matta 19 conducted an analysis of a national database of patients admitted to hospital in the United States from 2007 to 2009, covering 508,230 bariatric surgeries, and identified a 1.3% incidence of DVT and a 0.9% incidence of PTE, with PTE mortality rates of 3.9% among those who had vena cava filters fitted and 2.7% among those who did not. 

 With relation to prevention of thromboembolic events, the American College of Chest Physicians recommends mechanical prophylaxis for low risk patients, preferably intermittent pneumatic compression. For moderate risk patients with no risk of bleeding, the recommendation is LWMH or unfractionated heparin or mechanical prophylaxis (intermittent pneumatic compression). For high risk patients with no risk of bleeding, prophylaxis is achieved with LWMH or unfractionated heparin, in combination with intermittent pneumatic compression or elastic stockings. If heparin is contradicted and there is low risk of bleeding, it is recommended that low doses of aspirin, fondaparinux or mechanical prophylaxis (intermittent pneumatic compression) be used. 10

 In our study, patients had been stratified as at high risk of developing DVT and therefore prophylaxis with enoxaparin at 40 mg per day was chosen. However, the major weight gain among bariatric surgery patients raises the question of whether the LWMH dose recommended in the literature might be underestimated and if there could possibly be a need for higher doses of LWMH in this population, with a possible higher risk of preoperative or postoperative bleeding or of other complications. 

 Scholten et al. compared different doses of enoxaparin (30 mg vs. 40 mg every 12h) in 481 bariatric surgery patients and identified a 5.4% DVT rate in the group given the lower dose (30 mg) and 0.6% in the higher dose group (40 mg), although the duration of the surgical procedure and the length of hospital stay were both longer in the lower dose (30 mg) group. 20 Borkgren-Okonek et al. 21 used higher DVT prophylaxis doses in 223 gastric bypass patients, administering 40 mg every 12h to patients with BMI < 50 and 60 mg every 12h to patients with BMI over 50, and did not observe significant differences in DVT (0.45%) or bleeding (2.2%) rates. 

 Comparison of the use of different doses of LWMH for DVT prophylaxis in our study showed that, for the population investigated, there was zero incidence of DVT (in the femoropopliteal and popliteo-pedal segments or in the gastrocnemius and soleal muscle veins) in either of the groups. However, it should be pointed out that, in addition to pharmaceutical prophylaxis and early mobilization, all of the patients wore pneumatic boots for 24h and antithrombotic elastic stockings (18 mmHg) for 10 days during the postoperative period. These additional measures could have had an influence on the results, but using them together with the standard LWMH dose for the patients’ risk level was sufficient to avoid DVT during the postoperative period. 

 With relation to intraoperative and postoperative complications, there was no significant intraoperative bleeding in the CG, whereas in the SG there were two cases of minimal bleeding along the staple line of the excluded stomach, which was successfully controlled by the tie-over suturing that is routinely performed. Although the bleeding was of minimal volume and easily controlled, the fact that it only occurred in the SG could be related to the larger dose of LWMH and could therefore be considered an intraoperative complication, with a possibility of greater risk of bleeding case if higher doses of de LWMH are chosen for DVT prophylaxis. There was no need to abandon videolaparoscopic surgery in favor of conventional surgery because of bleeding or other intraoperative complications in either group. 

 During the postoperative period, two patients exhibited melena in each group. This may be a natural occurrence caused by residual blood after stapling of the stomach and intestine, in addition to the anastomoses, causing minor intraluminal bleeding that is expelled in the form of coagulated blood mixed with feces. Additionally, anticoagulants could also be the cause of intraluminal bleeding that manifests in this manner during the postoperative period and is generally self-limiting. 

 With regard to the possibility that there could be a higher risk of bleeding in the SG than in the CG because of higher doses of LWMH, the comparisons of anti-Xa factor, PTT, PT and platelet levels did not reveal statistically significant differences between the groups, even though there were more patients with BMI > 40 in the SG . 

 Heparin is an anticoagulant composed of polysaccharide chains of different molecular weights with a great affinity for antithrombin (AT), the physiological coagulation inhibitor, especially thrombin (IIa) and activated factor X (Xa). Low molecular weight heparins maintain the anti-Xa activity, but have reduced anti-IIa activity. Monitoring anti-Xa factor levels is one option for control of prophylaxis, although its utility has not yet been universally defined and controlled studies do not demonstrate a clear relationship with bleeding or thromboembolic events. 15

 The recommendation is to measure peak anti-Xa activity approximately 4h after administration of subcutaneous LWMH, since each type of LWMH has different concentration levels. The anti-Xa actions of LWMHs have been measured in volunteers both for prophylaxis and for treatment and enoxaparin (the drug used in our study) has been tested in people weighing up to 120 kg. The majority of authors test anti-Xa factor level between the third and fifth day after surgery. 22

 Mean anti-Xa activity, measured 3 to 5 hours after subcutaneous injection, is proportional to the dose administered, corresponding to 0.2, 0.4, 1.0 and 1.3 UI anti-Xa/mL at single doses of 20, 40, 1 and 1.5 mg/kg, respectively. 23 ^-^ 25 Levine et al. demonstrated a statistical correlation between anti-Xa factor levels below 0.1 UI/mL and thromboembolic events. 26

 In our study, there was a weak correlation between anti-Xa factor level and BMI in both the CG and the SG. Anti-Xa factor levels were not significantly different between groups, varying from 0.2 to 0.7 (mean of 0.4) in the CG and from 0.1 to 0.7 (mean of 0.4) in the SG and mean body weight was less than 120 kg in both groups. 

 Therefore, we conclude that there was no statistically significant difference when higher doses of LWMH were used for DVT prophylaxis in patients who were candidates for bariatric surgery in terms of risk of DVT, anti-Xa factor levels, or preoperative and postoperative bleeding.
